# Negative Effects of Mobile Phone Addiction Tendency on Spontaneous Brain Microstates: Evidence From Resting-State EEG

**DOI:** 10.3389/fnhum.2021.636504

**Published:** 2021-04-28

**Authors:** Hao Li, Jingyi Yue, Yufeng Wang, Feng Zou, Meng Zhang, Xin Wu

**Affiliations:** School of Psychology, Xinxiang Medical University, Xinxiang, China

**Keywords:** mobile phone addiction, resting-state EEG, microstates, mobile phone addiction tendency, brain function

## Abstract

The prevalence of mobile phone addiction (MPA) has increased rapidly in recent years, and it has had a certain negative impact on emotions (e.g., anxiety and depression) and cognitive capacities (e.g., executive control and working memory). At the level of neural circuits, the continued increase in activity in the brain regions associated with addiction leads to neural adaptations and structural changes. At present, the spontaneous brain microstates that could be negatively influenced by MPA are unclear. In this study, the temporal characteristics of four resting-state electroencephalogram (RS-EEG) microstates (MS1, MS2, MS3, and MS4) related to mobile phone addiction tendency (MPAT) were investigated using the Mobile Phone Addiction Tendency Scale (MPATS). We attempted to analyze the correlation between MPAT and corresponding microstates and provide evidence to explain the brain and behavioral changes caused by MPA. The results showed that the total score of the MPATS was positively correlated with the duration of MS1, related to phonological processing and negatively correlated with the duration of MS2, related to visual or imagery processing, and MS4, related to the attentional network; the score of the withdrawal symptoms subscale was additionally associated with duration of MS3, related to the cingulo-opercular emotional network. Based on these results, we believe that MPAT may have some negative effects on attentional networks and sensory brain networks; moreover, withdrawal symptoms may induce some negative emotions.

## Introduction

With the multiple and ever-changing functions of mobile phones, internet use and mobile phone use have become closely interwoven (Montag et al., 2015). In China, by the end of June 2019, the Internet penetration rate had reached 61.2%, and 99.1% of Internet users preferred to use their mobile phones to access the Internet (CNNIC, 2019). Among college students in China, for instance, the penetration rate of mobile phones rose from 84.6 to 99.3% between 2012 and 2015 (Bian, 2015; Long et al., 2016), and it is continually rising, while the prevalence of mobile phone dependence has been found to range from 4.1 to 37.9% (Wang and Zhang, 2015; Chen et al., 2016; Long et al., 2016). Mobile phone addiction (MPA) refers to individuals whose mobile phone use behavior is out of control, resulting in a state of obsession, which can be categorized as a problematic behavior (Salehan and Negahban, 2013). An immediate impact on college students is that a higher level of MPA leads to a decline in their academic performance (Soyemi et al., 2015). Additionally, Jacobsen and Forste (2011) identified a significant negative association between the use of mobile phones and academic performance among first-year university students in the United States. MPA has become a global concern because of its negative effects on memory and interpersonal communication (Hao et al., 2019; Miri et al., 2019), as well as its association with negative emotions (anxiety, depression, stress, and loneliness) (Demirci et al., 2015; Chen et al., 2016; Gao et al., 2018).

According to current research, MPA has a significant negative impact on executive function. Addicts to certain online apps that involve communication characteristics show more social anxiety, emotional deficits, and impaired prefrontal cortex-related inhibitory control (Dieter et al., 2017). A significant positive correlation was found between the number of errors in the Stroop task and the short-version Smartphone Addiction Scale score (Kwon et al., 2013). These findings may reflect the exact relationship between MPA and inhibitory control processes (Dilce et al., 2017). Furthermore, negative associations have been found between MPA and working memory (Billieux et al., 2008), executive function (Billieux, 2012), self-control, and self-monitoring (Takao et al., 2009). Several neuroimaging studies have provided compelling evidence for behavioral and neurobiological similarities and correlations between different types of addictions, hypothesizing that there is a fundamentally identical neural mechanism (Bianchi and Phillips, 2005; Billieux et al., 2015; Zhang and Liu, 2017). A better understanding of MPA and its underlying mechanisms may also reveal other types of addiction, and vice versa (Billieux et al., 2015; Zhang and Liu, 2017). Individuals with behavioral addiction are often characterized as exhibiting abnormal function in brain regions that include the prefrontal cortex, anterior cingulate cortex (ACC) (Grant et al., 2010), ventral striatum (Han et al., 2012), insula (Kuss and Griffiths, 2012), and thalamus (Ruth et al., 2010). It is worth noting that altered brain morphology in these areas has also been reported in Internet addicts as well as gambling addicts (Wang and Zhang, 2015). This provides morphological evidence of structural changes in the brain of individuals with MPA, for which we will further explore the corresponding functional changes through resting-state electroencephalogram (RS-EEG). At the level of neural circuits, the continued increase in the activity of brain regions associated with addiction leads to neural adaptations and structural changes (Kuss and Griffiths, 2012). This process is undoubtedly slow and long-lasting. Hence, we explore the changes in brain activity caused by MPA from the perspective of mobile phone addiction tendency (MPAT).

The electroencephalogram (EEG) is a widely used non-invasive tool for measuring the electrical physiology of the brain (Ingber and Nunez, 2011) that can detect and record millivolt fluctuations of cortical potential with very high temporal resolution and make it easier to assess dynamically changing mental activities (Canuet et al., 2012; Mani et al., 2013; Nishida et al., 2013). RS-EEG microstates are a method that defines the states of the multichannel EEG signals by the spatial topographies of electric potentials over the electrode array (Norbaidurah et al., 2018). Previous studies revealed that four prototypical microstates (MS1, MS2, MS3, and MS4) explain nearly 70–80% of the variance of EEG brain activity during wakeful rest (Seitzman et al., 2017; Norbaidurah et al., 2018). Moreover, it has been found that these four RS-EEG microstates are related to certain brain networks. MS1 is correlated with activations primarily in the bilateral superior and middle temporal gyri, which are implicated in phonological processing and also involved in speech and auditory processing or auditory (Britz et al., 2010; Seitzman et al., 2017). MS2 is correlated with bilateral extravasate visual areas (BA18 and BA19), which have been identified as the visual network (Damoiseaux et al., 2006; Mantini et al., 2007). MS3 is correlated with activations in the dorsal anterior cingulate cortex (dACC), the bilateral inferior frontal cortices, and the insula, which are related to the saliency network (SN) (Fox et al., 2006; Seeley et al., 2007) and play a critical role in switching between central executive function and the default mode (Sridharan et al., 2008). MS4 is correlated with signaling in the right-lateralized dorsal and ventral areas of the frontal and parietal cortex, which are related to ventral fronto-parietal attentional networks and are associated with switching and reorientation of attention (Corbetta and Shulman, 2002).

Considering the high time resolution of EEG, RS-EEG microstates can also reflect the dynamic characteristics of these brain networks, such as duration (the stability of underlying neural assemblies for a certain microstate), occurrence (neural generators that become activated for a certain microstate), coverage (the time coverage for a certain microstate relative to others), as well as the possibility of transition between any two RS-EEG microstates (Lehmann et al., 2005; Khanna et al., 2015). Moreover, the characteristics are also associated with the altered mental states under experimental conditions. Seitzman et al. (2017) found that that the duration, coverage, and occurrence of MS4 were significantly higher during the cognitive task compared to wakeful rest, while MS3 showing significantly decreased. Furthermore, MS2 and MS3 were altered by manipulations of visual input, with increased occurrence in the eyes open condition. Zappasodi et al. (2017) also found that MS2 and MS3 were regulated by visuospatial tasks, reflecting that the contribution of MS2 significantly increased while the contribution of MS3 significantly decreased under visuospatial tasks. In addition, during the eyes open condition, MS1 and MS4 had significantly shorter durations, while MS3 had increased occurrence. MS4 had decreased coverage in the eyes open condition (Seitzman et al., 2017). Croce et al. (2020) observed that as the amplitude of alpha oscillations within the subject increased, the parameters of MS2 increased, the coverage of MS4 decreased, and the frequency of MS3 increased. Research has demonstrated that task-related microstates would re-emerge during post-task periods of rest (Murphy et al., 2018). In other words, the resting-state microstate will be affected by previous activity. Different microstates reflect different neural network activities and thus reflect different cognitive processes or mental states (Croce et al., 2020). Microstate parameters correspond to the dynamic characteristics of microstates or brain networks (Khanna et al., 2015). The microstate time series in the resting state EEG represents the rapid switch between the activities of various neurons in the brain in the resting state. Resting-state EEG microstate parameters can be used as objective neurophysiological and biological indicators to provide a method for monitoring disease or other activities (Khanna et al., 2015).

In this study, based on the negative effects of MPA on executive control and emotion, we examined the influence of MPAT on the spontaneous brain activities related to executive control and the generation of emotions. The negative effects of MPAT on the temporal characteristics of the four RS-EEG microstates were investigated by using the Mobile Phone Addiction Tendency Scale (MPATS) to measure MPA. We hypothesized that MS4, related to the executive function, and MS2, related to visual processing, would be affected. Additionally, the activation of the dACC, insula, and inferior frontal gyrus has been found to increase significantly under negative emotions (Tolle et al., 1999; Coen et al., 2009; Harlé et al., 2012). Meanwhile, withdrawal symptoms are defined as a negative physical or psychological reaction to not using a mobile phone and are attributed to anhedonia, whose main manifestation is mood change (Zhang, 2006), such as intense anxiety. Hence, we hypothesized that withdrawal symptoms might be related to mood-related MS3.

## Materials and Methods

### Subjects

The sample consisted of 335 undergraduate students (27.2% male) from the Xinxiang Medical University (*M* = 18.3, SD = 0.84, range: 18–22 years). We screened out 53 participants who had not completed EEG experiments or had incomplete data. Participants were asked not to take the drug for several days before the experiment. All experiments were conducted with the understanding and written informed consent of each participant, which was in accordance with the Declaration of Helsinki. The protocol was approved by the Ethics Committee of the Xinxiang Medical University. Any question from the participant was clarified.

### Mobile Phone Addiction Tendency Scale (MPATS)

This scale, developed by Xiong et al. (2012), referred to the existing research results on mobile phones, and, according to the actual situation of college students, through interviews, predictions, and formal tests, a formal scale with a good representative item was finally determined. The scale consists of 16 items grouped into four factors: withdrawal symptoms (negative physical or psychological reactions to not participating in mobile phone activity), salience behavior (the use of mobile phones occupies the center of thought and action), social comfort (the role of mobile phone in interpersonal communication), and mood changes (changes in mood caused by mobile phones). Using a five-point Likert scale, the scores ranged from 1 to 5 points, i.e., “very inconsistent” to “very consistent,” respectively. The higher the total score, the more serious the addiction tendency is. The Cronbach coefficient of the total scale was 0.83, and the Cronbach coefficients of withdrawal symptoms, highlight behavior, social comfort, and mood change four factors were 0.80, 0.64, 0.68, and 0.55, respectively. In our samples, the Cronbach coefficients of the total scale were 0.87, and the Cronbach coefficients of the withdrawal symptoms, salient behaviors, social comfort, and mood changes were 0.80, 0.70, 0.82, and 0.40, respectively.

### RS-EEG Data Acquisition

All participants participated in data collection in an EEG lab that required low light and quiet. The RS-EEG recording was about 6 min. During the collection process, subjects were asked to relax, close their eyes, and enter a resting state to avoid swallowing, blinking, and other activities that may cause artifacts. Data were collected using instrument Cerebus 128TM system (Cyberkinetics, United States). EEG data were recorded from 64 Ag-AgCl scalp sites according to the international 10–20 system in an elastic cap (NeuroScan Product). During recording, all electrodes were referenced to Cz and re-referenced off-line to linked mastoids. Channels for horizontal and vertical EOG were computed offline from electrodes recorded from the outer canthi of the eyes and from above and below the right eye, respectively. The impedance between the electrodes and the participant’s scalp was kept below 10 kΩ.

### RS-EEG Microstate Pre-processing

The raw data files from the EGI were transformed into the MAT file format for pre-processing using the EEGLAB^1^ v13.0.0 toolbox. EEG was sampled online with 500 Hz frequency DC amplifiers with a band-pass filter of 2–20 Hz (West et al., 2008). Artifacts produced by blinks or eye movements were corrected by subtracting the means of ICAs (Koenig et al., 1999, 2002; Lehmann et al., 2005) implemented in the EEGLAB software. The artifact-free data were recomputed against the average according to previous studies (Pascual-Marqui et al., 1995; Naqvi et al., 2007; Etkin et al., 2011; Harlé et al., 2012) and was average re-referenced. Then the data were segmented into 180 epochs with an epoch length of 2000 ms.

### RS-EEG Microstates Analysis

**FIGURE 1:**
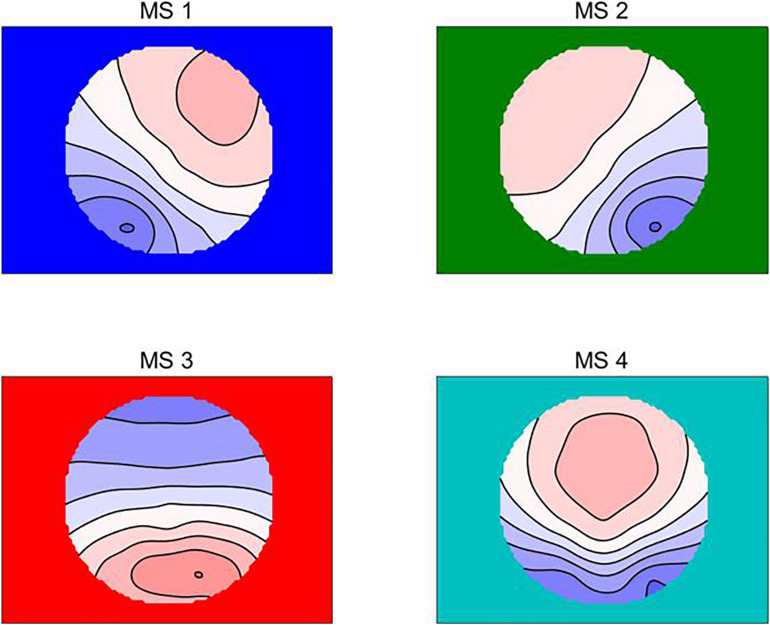
The four microstate topographic maps are RS-EEG microstate Type A (MS1), Type B (MS2), Type C (MS3), and Type D (MS4) (Wu et al., 2020).

First, the global field power (GFP) was calculated using the selected EEG epoch (Cai et al., 2018). After that, based on previous studies (Tibshirani and Walther, 2005), the Atomize-Agglomerate Hierarchical cluster (AAHC) were used to analyze the microstates with the polarity of each topographical map being disregarded. The AAHC was a modified k-means to provide unique clusters for microstate analysis (Murray et al., 2008). Third, the cross-validation criterion is used to determine the optimal cluster number, that is, the optimal cluster number can find the least template mapping, and the global interpretation variance is the largest (Damoiseaux et al., 2006; Schlegel et al., 2011). According to our data, four clusters were found, and the explained variance was 0.787 ± 0.033. Lehmann and his colleagues labeled them A, B, C, and D, while we used MS1,MS2, MS3, and MS4 (Lehmann et al., 2007; Seitzman et al., 2017; Norbaidurah et al., 2018; Figure 1). Raw data were then fitted according to global map dissimilarity (GMD), and each time point was labeled as the cluster chart with the best correlation (Gao et al., 2017).

For each microstate class, the following parameters were calculated: mean duration, i.e., the mean time (in ms) reflecting the stability of its underlying neural assemblies; mean occurrences per second across all analysis epochs; contribution, i.e., mean percentage of time covered by each microstate class across analysis epochs (summing up to 100% across all four microstate classes); and the non-random transition probabilities from each microstate to another, which are often interpreted to suggest an encoded sequential activation of the neural assemblies that generate microstates (Lehmann et al., 2005; Andreou et al., 2014; Khanna et al., 2015).

### Statistical Analysis

Data were analyzed using SPSS software (22.0), and the scores of each scale were described and statistically analyzed. The normality of the distributions was tested using the Shapiro–Wilk test. Some variables did not conform to the normal distribution; we normalized these variables for further statistical analysis. Pearson correlation analysis was used to explore the correlation between MPA tendency and microstate composition and transition. For the level of statistical significance, we set *p* ≤ 0.01. *Post hoc* comparisons on the unstandardized residuals were considered significant at *p* < 0.0125 (e.g., *p* < 0.05, with Bonferroni correction for comparing across the four microstates). In all of these statistical analyses, age and sex were seen as covariates.

## Results

### Behavioral Results

Normalization to the four variables, such as withdrawal symptoms, salience behavior, social comfort, and mood changes, have come to nothing, but they all had kurtosis and skewness of less than 1. Therefore, we regarded them as an approximate normal distribution for further statistical analysis (Table 1).

**TABLE 1:** Behavioral results of MPATS (*n* = 335).

	Mean	SD	Normal distribution test
Withdrawal symptoms	17.84	4.69	Approximately normal distribution
Salience behavior	9.35	3.01	Approximately normal distribution
Social comfort	8.40	2.88	Approximately normal distribution
Mood changes	7.47	2.30	Approximately normal distribution
The total score	43.06	10.31	Normal distribution

### The Relationship Between Questionnaire Scores and Microstates

We did not detect correlations between coverage and the MPATS’ four dimensions or the total score. According to the correlation analysis, withdrawal symptoms were significantly positively correlated with the duration of MS1 and of MS3 (*r* = 0.164, *p* = 0.003; *r* = 0.146, *p* = 0.007, respectively) and were significantly negatively correlated with the occurrence of MS2 and of MS4 (*r* = −0.152, *p* = 0.005; *r* = 0.178, *p* = 0.001, respectively). The total score was also significantly positively correlated with the duration of MS1 (*r* = 0.159, *p* = 0.003) and significantly negatively correlated with the occurrence of MS2 and of MS4 (*r* = −0.153, *p* = 0.005; *r* = −0.155, *p* = 0.004, respectively) (Table 2). No correlation was found between microstate transition and MPATS scores (Table 3).

**TABLE 2:** Correlations for Microstates components and MPAT.

	Withdrawal symptoms	Salience behavior	Social comfort	Mood changes	The total score
**Duration**					
MS 1	***0.173***	0.092	0.136	0.096	***0.165***
MS 2	0.098	0.002	0.070	0.038	0.073
MS 3	***0.149***	0.069	0.083	0.099	0.133
MS 4	0.073	0.033	0.083	0.022	0.071
**Occurrence**					
MS 1	−0.049	−0.000	−0.024	−0.020	−0.033
MS 2	***−0.155***	−0.101	−0.102	−0.108	−***0.152***
MS 3	−0.080	0.002	−0.140	−0.033	−0.082
MS 4	−***0.179***	−0.068	−0.088	−0.130	−***0.0154***
**Coverage**					
MS 1	0.085	0.066	0.089	0.056	0.096
MS 2	−0.081	−0.109	−0.054	−0.086	−0.103
MS 3	0.059	0.039	−0.053	0.072	0.040
MS 4	−0.088	−0.022	0.006	−0.074	−0.062

*The bolded and italic values meant that the relationship was significant at *p* < 0.01 with Bonferroni correction.*

**TABLE 3:** Correlations for Microstates transitions and MPAT.

	Withdrawal symptoms	Salience behavior	Social comfort	Mood changes	The total score
**Transition**					
MS1 to MS2	0.003	−0.030	0.030	−0.027	−0.005
MS1 to MS3	0.133	0.104	−0.005	0.138	0.120
MS1 to MS4	−0.001	0.021	0.105	−0.007	0.033
MS2 to MS1	−0.007	−0.030	0.023	−0.033	−0.013
MS2 to MS3	−0.002	−0.035	−0.053	−0.002	−0.026
MS2 to MS4	−0.112	−0.097	−0.042	−0.091	−0.111
MS3 to MS1	0.134	0.103	−0.018	0.130	0.115
MS3 to MS2	−0.016	−0.045	−0.041	0.005	−0.031
MS3 to MS4	−0.043	0.018	−0.049	−0.041	−0.037
MS4 to MS1	0.003	0.017	0.108	−0.004	0.036
MS4 to MS2	−0.117	−0.081	−0.060	−0.970	−0.115
MS4 to MS3	−0.037	0.011	−0.043	−0.043	−0.035

## Discussion

The purpose of this study was to use microstates to determine the relationship between college students’ MPAT and changes in brain function using RS-EEG microstates. The results showed that withdrawal symptoms are significantly positively correlated with the duration of MS3, and the total score of the MPATS is significantly negatively correlated the with the occurrence of MS2 and MS4. We unexpectedly found that the total score had a significant positive correlation with MS1.

With regard to the correlation between withdrawal symptoms and the MS3 microstate, the idea that insula dysfunction underlies drug addiction is supported by a study showing that chronic cocaine users have reduced gray:white matter ratios in the insula (Franklin et al., 2002). In one case, a patient with insula injury claimed that his body had forgotten the urge to smoke (Naqvi et al., 2007). It has been found that MS3 is positively BOLD related to the fronto-insular SN, including the dACC, the bilateral inferior frontal cortex, and the insula (Fox et al., 2006; Seeley et al., 2007), which play a critical role in switching between central executive function and the default mode (Sridharan et al., 2008). The experiments of Miri et al. (2019) showed that the physical and psychological effects of excessive cell phone use include headaches and memory loss, and the brain regions involved in the default mode network (MS3) include the hippocampus, which explains why memory loss occurs in addicts. From the above evidence, we may infer that the addict’s insula activity increases, leading to withdrawal. Activation of the dACC, insula, and inferior frontal gyrus has been found to increase significantly under negative emotions (Tolle et al., 1999; Coen et al., 2009; Harlé et al., 2012). The dACC region is involved in the assessment and expression of negative emotions (Etkin et al., 2011), such as fear (Perlman and Pelphrey, 2010) and anxiety (Afif et al., 2010). The dACC is closely related to the attention distribution of emotional information; that is, the more strongly a person is aware of the characteristics of his own emotional experience, the higher the degree of dACC participation in the process of emotional arousal (McRae et al., 2008). The insula is also important for emotional feelings (Gasquoine, 2014). Consistent with our hypothesis, we found that withdrawal symptoms are related to MS3. We can speculate that the change in MS3 reflects the sensitivity of mobile phone addicts to negative emotions or their degree of attention to negative emotional experiences.

Furthermore, the total score on the MPATS was significantly negatively correlated with the occurrence of MS4. MS4 is related to the dorsal attention functional system and is associated with switching and reorientation of attention (Corbetta and Shulman, 2002). Precious topographical analyses indicated that the duration, coverage, and occurrence of MS4 were significantly higher during cognitive tasks compared to wakeful rest (Seitzman et al., 2017). Although our experiment reflected occurrence only, it was consistent with decreased cognitive activity in mobile phone addicts. In addition, there is a strong link between working memory and selective attention (Ming and Yang, 2007). For example, the contents of working memory are related to the orientation of selective attention, and selective attention is involved in the maintenance and updating of information in working memory (LaBar et al., 1999; Awh and Jonides, 2001). Based on previous research, individuals with Internet addiction present deficiencies in working memory (Zhou et al., 2015). The potential similarities in behavioral and neurobiological factors between MPA and Internet addiction (Billieux et al., 2015; Wang et al., 2016), combined with our findings, suggest that both play the same role in working memory and attention. In short, our findings show deficits in attention shifting and redirecting and working memory in mobile phone addicts. It is worth mentioning that some studies have found that MPA is beneficial to cognition. For example, in a study of college students, Zhang and Liu found that the MPA group had better attention conversion and cognitive flexibility than the non-addicted group (Zhang and Liu, 2017). Other researchers found that Internet addicts were more sensitive to exogenous stimuli, leading to an improvement in their attention function (West et al., 2008). Some studies have shown that addicts can quickly identify visual stimuli associated with a task and divert attention from stimuli that are irrelevant, and they are better able to recover from captivity when they realize they have a clue (Li et al., 2011).

Finally, the total score on the MPATS was positively correlated with the duration of MS1. Previous studies found that MS1 was negatively BOLD associated with activation of the bilateral superior and middle temporal gyri, which may imply that individuals with a short duration of MS1 possess a stronger function of phonological processing (Damoiseaux et al., 2006; Britz et al., 2010). Research also suggests a link between the overuse of mobile phones and hearing problems (Meo and Al-Dreess, 2005). Therefore, we can infer that mobile phone addicts also have worse auditory information processing and speech processing ability than healthy controls. This could be a future research direction. In our study, we found that the (auditory-associated) perception system was affected, which we did not expect. We know that perception of inter-parental conflict affects Internet addiction directly and indirectly (Zheng and Deng, 2015). We can guess that mobile phone addicts show the same pattern. Our experiment also confirmed visual influence (MS2 was negatively correlated with the scale score).

However, our study has some limitations. First, the results only showed weak correlations, which may be due to the large sample size in this study. In addition, all subjects with complete data were selected for correlation analysis in this study. If we selected only high and low subgroups (for example, 27%) based on the score division, the results may be more meaningful because significant inter-group differences may be obtained. Second, our experimental results were only based on the combined analysis of existing experiments and literature, and their complete accuracy cannot be guaranteed. Third, we did not further differentiate the studies on the related brain regions of MS3 and MS4, which are relatively complex microstate types, and this should be explored in future research. Unlike what we expected, we did not find a correlation between the MPATS score and MA3. MS3 is mainly related to the SN (Fox et al., 2006; Seeley et al., 2007). Evidence from a large number of brain imaging studies across multiple task domains suggests that the anterior insula and ACC nodes of SN respond to degrees of subjective salience, whether related to cognition, homeostasis, or mood (Craig, 2009; Vinod and Uddin, 2010). This is because the insula is important for emotional feelings (Gasquoine, 2014) and is related to withdrawal symptoms (Franklin et al., 2002). In addition, other addiction studies have found that the insula structure changes in addicts (Franklin et al., 2002), so at the beginning of the study, we speculated that MS3 was related to the MPATS score. But in fact, our subjects are normal college students, and there are no extreme mobile phone addicts, so there may not be significant structural changes. SN is responsible for regulating attention based on various information, such as the physical properties of the stimulus or its relevance to the task at hand, and is responsible for judging the salience of the stimulus and regulating attention (Menon, 2015; Chen et al., 2017). However, we collected resting-state data, so no significant correlation was obtained.

## Data Availability Statement

The raw data supporting the conclusions of this article will be made available by the authors, without undue reservation.

## Ethics Statement

The studies involving human participants were reviewed and approved by the Ethics Committee of the Xinxiang Medical University. The participants provided their written informed consent to participate in this study.

## Author Contributions

XW, JY, and HL designed the research, wrote the manuscript, and analyzed the data. FZ, MZ, and YW collected the data. All authors contributed to the article and approved the submitted version.

## Conflict of Interest

The authors declare that the research was conducted in the absence of any commercial or financial relationships that could be construed as a potential conflict of interest.

## Footnotes

1 https://sccn.ucsd.edu/eeglab/index.php

## References

[B1] Afif A. Minotti L. Kahane P. Hoffmann D. (2010). Anatomofunctional organization of the insular cortex: a study using intracerebral electrical stimulation in epileptic patients. *Epilepsia* 51 2305–2315. 10.1111/j.1528-1167.2010.02755.x 20946128

[B2] Andreou C. Faber P. L. Leicht G. Schoettle D. Polomac N. Hanganu-Opatz I. L. (2014). Resting-state connectivity in the prodromal phase of schizophrenia: insights from EEG microstates. *Schizophrenia Res.* 152 513–520. 10.1016/j.schres.2013.12.008 24389056

[B3] Awh E. Jonides J. (2001). Overlapping mechanisms of attention and spatial working memory. *Trends Cogn. Sci.* 5 119–126. 10.1016/s1364-6613(00)01593-x 11239812

[B4] Bian L. (2015). LeungLinking loneliness, shyness, smartphone addiction symptoms, and patterns of smartphone use to social capital. *Soc. Sci. Comput. Rev.* 33 61–79. 10.1177/0894439314528779

[B5] Bianchi A. Phillips J. G. (2005). Psychological predictors of problem mobile phone use. *CyberPsychol. Behav.* 8 39–51. 10.1089/cpb.2005.8.39 15738692

[B6] Billieux J. (2012). Problematic use of the mobile phone: a literature review and a pathways model. *Curr. Psychiatry Rev.* 8 299–307. 10.2174/157340012803520522

[B7] Billieux J. Maurage P. Lopez-Fernandez O. Kuss D. J. Griffiths M. D. (2015). Can disordered mobile phone use be considered a behavioral addiction? An update on current evidence and a comprehensive model for future research. *Curr. Addiction Rep.* 2 156–162. 10.1007/s40429-015-0054-y

[B8] Billieux J. Van der Linden M. Rochat L. (2008). The role of impulsivity in actual and problematic use of the mobile phone. *Appl. Cogn. Psychol.* 22 1195–1210. 10.1002/acp.1429

[B9] Britz J. Van De Ville D. Michel C. M. (2010). BOLD correlates of EEG topography reveal rapid resting-state network dynamics. *Neuroimage* 52 1162–1170. 10.1016/j.neuroimage.2010.02.052 20188188

[B10] Cai Y. Huang D. Chen Y. Yang H. Wang C. Zhao F. (2018). Deviant dynamics of resting state electroencephalogram microstate in patients with subjective tinnitus. *Front. Behav. Neurosci.* 12:122. 10.3389/fnbeh.2018.00122 29988458 PMC6024160

[B11] Canuet L. Tellado I. Couceiro V. Fraile C. Fernandez-Novoa L. Ishii R. (2012). Resting-state network disruption and APOE genotype in Alzheimer’s disease: a lagged functional connectivity study. *PLoS One* 7:e46289. 10.1371/journal.pone.0046289 23050006 PMC3457973

[B12] Chen J.-J. Liu J.-R. Wei X. Li Y.-B. Zhu J. Li W. (2017). The abnormal salience network of the brain in heroin addicts:a resting-state functional magnetic resonance image study based on independent component analysis. *Magnetic Resonance Imaging* 2017 100–104.

[B13] Chen L. Yan Z. Tang W. Yang F. Xie X. He J. (2016). Mobile phone addiction levels and negative emotions among Chinese young adults: the mediating role of interpersonal problems. *Comput. Hum. Behav.* 55 856–866. 10.1016/j.chb.2015.10.030

[B14] CNNIC (2019). *The 44th China Statistical Report on Internet Development.* Available online at: http://www.cnnic.net.cn/hlwfzyj/hlwxzbg/hlwtjbg/201908/t20190830_70800.htm (accessed August 30, 2019).

[B15] Coen S. J. Yágüez L. Aziz Q. Mitterschiffthaler M. T. Brammer M. Williams S. C. R. (2009). Negative mood affects brain processing of visceral sensation. *Gastroenterology* 137 253.e–261.e. 10.1053/j.gastro.2009.02.052 19582887

[B16] Corbetta M. Shulman G. L. (2002). Control of Goal-directed and Stimulus-driven attention in the brain. *Nat. Rev. Neurosci.* 3 201–215. 10.1038/nrn755 11994752

[B17] Craig A. D. (2009). How do you feel—now? The anterior insula and human awareness. *Nat. Rev. Neurosci.* 10 59–70. 10.1038/nrn2555 19096369

[B18] Croce P. Quercia A. Costa S. Zappasodi F. (2020). EEG microstates associated with intra- and inter-subject alpha variability. *Sci. Rep.* 10:2469. 10.1038/s41598-020-58787-w 32051420 PMC7015936

[B19] Damoiseaux J. S. Rombouts S. A. R. B. Barkhof F. Scheltens P. Stam C. J. Smith S. M. (2006). Consistent resting-state networks across healthy subjects. *Proc. Natl. Acad. Sci. U.S.A.* 103 13848–13853. 10.1073/pnas.0601417103 16945915 PMC1564249

[B20] Demirci K. Akgönül M. Akpinar A. (2015). Relationship of smartphone use severity with sleep quality, depression, and anxiety in university students. *J. Behav. Addict.* 4 85–92. 10.1556/2006.4.2015.010 26132913 PMC4500888

[B21] Dieter J. Hoffmann S. Mier D. Reinhard I. Beutel M. Vollstädt-Klein S. (2017). The role of emotional inhibitory control in specific internet addiction – an fMRI study. *Behav. Brain Res.* 324 1–14. 10.1016/j.bbr.2017.01.046 28174031

[B22] Dilce T. Sena B. Sena N. G. (2017). A new eye-gazing behavioral task for smartphone addiction in relation to inhibitory control and its validation via stroop task. *Koç Univ. Undergraduate Psychol. J.* 2018 9–15.

[B23] Etkin A. Egner T. Kalisch R. (2011). Emotional processing in anterior cingulate and medial prefrontal cortex. *Trends Cogn. Sci.* 15 85–93. 10.1016/j.tics.2010.11.004 21167765 PMC3035157

[B24] Fox M. D. Corbetta M. Snyder A. Z. Vincent J. L. Raichle M. E. (2006). Spontaneous neuronal activity distinguishes human dorsal and ventral attention systems. *Proc. Natl. Acad. Sci. U.S.A.* 103 10046–10051. 10.1073/pnas.0604187103 16788060 PMC1480402

[B25] Franklin T. R. Acton P. D. Maldjian J. A. Gray J. D. Croft J. R. Dackis C. A. (2002). Decreased gray matter concentration in the insular, orbitofrontal, cingulate, and temporal cortices of cocaine patients. *Biol. Psychiatry* 51 134–142. 10.1016/s0006-3223(01)01269-0 11822992

[B26] Gao T. Li J. Zhang H. Gao J. Kong Y. Hu Y. (2018). The influence of alexithymia on mobile phone addiction: the role of depression, anxiety and stress. *J. Affective Disord.* 225 761–766. 10.1016/j.jad.2017.08.020 28926906

[B27] Gao Z. K. Cai Q. Yang Y. X. Dong N. Zhang S. S. (2017). Visibility graph from adaptive optimal kernel time-frequency representation for classification of epileptiform EEG. *Int. J. Neural. Syst.* 27:1750005. 10.1142/s0129065717500058 27832712

[B28] Gasquoine P. G. (2014). Contributions of the insula to cognition and emotion. *Neuropsychol. Rev.* 24 77–87. 10.1007/s11065-014-9246-9 24442602

[B29] Grant J. E. Potenza M. N. Weinstein A. Gorelick D. A. (2010). Introduction to behavioral addictions. *Am. J. Drug Alcohol Abuse* 36 233–241. 20560821 10.3109/00952990.2010.491884 PMC3164585

[B30] Han D. H. Kim S. M. Lee Y. S. Renshaw P. F. (2012). The effect of family therapy on the changes in the severity of on-line game play and brain activity in adolescents with on-line game addiction. *Psychiatry Res. Neuroimag.* 202 126–131. 10.1016/j.pscychresns.2012.02.011 22698763 PMC4651430

[B31] Hao Z. Jin L. Li Y. Akram H. R. Saeed M. F. Ma J. (2019). Alexithymia and mobile phone addiction in Chinese undergraduate students: the roles of mobile phone use patterns. *Comput. Hum. Behav.* 97 51–59. 10.1016/j.chb.2019.03.001

[B32] Harlé K. M. Chang L. J. van ’t Wout M. Sanfey A. G. (2012). The neural mechanisms of affect infusion in social economic decision-making: a mediating role of the anterior insula. *NeuroImage* 61 32–40. 10.1016/j.neuroimage.2012.02.027 22374480

[B33] Ingber L. Nunez P. L. (2011). Neocortical dynamics at multiple scales: EEG standing waves, statistical mechanics, and physical analogs. *Math. Biosci.* 229 160–173. 10.1016/j.mbs.2010.12.003 21167841

[B34] Jacobsen W. C. Forste R. (2011). The wired generation: academic and social outcomes of electronic media use among university students. *Cyberpsychol. Behav. Soc. Netw.* 14 275–280. 10.1089/cyber.2010.0135 20961220

[B35] Khanna A. Pascual-Leone A. Michel C. M. Farzan F. (2015). Microstates in resting-state EEG: current status and futuredirections. *Neurosci. Biobehav. Rev.* 49 105–113. 10.1016/j.neubiorev.2014.12.010 25526823 PMC4305485

[B36] Koenig T. Lehmann D. Merlo M. C. G. Kochi K. Hell D. Koukkou M. (1999). A deviant eeg brain microstate in acute, neuroleptic-naive schizophrenics at rest. *Eur. Arch. Psychiatry Clin. Neurosci.* 249 205–211. 10.1007/s004060050088 10449596

[B37] Koenig T. Prichep L. Lehmann D. Sosa P. V. Braeker E. Kleinlogel H. (2002). Millisecond by millisecond, year by year: normative EEG microstates and developmental stages. *NeuroImage* 16 41–48. 10.1006/nimg.2002.1070 11969316

[B38] Kuss D. J. Griffiths M. D. (2012). Internet and gaming addiction: a systematic literature review of neuroimaging studies. *Brain Sci.* 2 347–374. 10.3390/brainsci2030347 24961198 PMC4061797

[B39] Kwon M. Kim D. J. Cho H. Yang S. (2013). The smartphone addiction scale: development and validation of a short version for adolescents. *PLoS One* 8:e83558. 10.1371/journal.pone.0083558 24391787 PMC3877074

[B40] LaBar K. S. Gitelman D. R. Parrish T. B. Mesulam M.-M. (1999). Neuroanatomic overlap of working memory and spatial attention networks: a functional MRI comparison within subjects. *NeuroImage* 10 695–704. 10.1006/nimg.1999.0503 10600415

[B41] Lehmann C. Koenig T. Jelic V. Prichep L. John R. E. Wahlund L.-O. (2007). Application and comparison of classification algorithms for recognition of Alzheimer’s disease in electrical brain activity (EEG). *J. Neurosci. Methods* 161 342–350. 10.1016/j.jneumeth.2006.10.023 17156848

[B42] Lehmann D. Faber P. L. Galderisi S. Herrmann W. M. Kinoshita T. Koenig T. (2005). EEG microstate duration and syntax in acute, medication-naïve, first-episode schizophrenia: a multi-center study. *Psychiatry Res.: Neuroimaging* 138 141–156. 10.1016/j.pscychresns.2004.05.007 15766637

[B43] Li X. Yang Y. Zhang Y. Zhang Q. Liu L. Du G. (2011). A behavioral study of attentional orientation in patients with online game addiction [J]. Chinese J. Behav. Med. Brain Sci. 20, 535–537.

[B44] Long J. Liu T.-Q. Liao Y.-H. Qi C. He H.-Y. Chen S.-B. (2016). Prevalence and correlates of problematic smartphone use in a large random sample of Chinese undergraduates. *BMC Psychiatry* 16:408. 10.1186/s12888-016-1083-3 27855666 PMC5114822

[B45] Mani A. Mullainathan S. Shafir E. Zhao J. (2013). Poverty impedes cognitive function. *Science* 341 976–980. 10.1126/science.1238041 23990553

[B46] Mantini D. Perrucci M. G. Del Gratta C. Romani G. L. Corbetta M. (2007). Electrophysiological signatures of resting state networks in the human brain. *Proc. Natl. Acad. Sci. U.S.A.* 104 13170–13175. 10.1073/pnas.0700668104 17670949 PMC1941820

[B47] McRae K. Reiman E. M. Fort C. L. Chen K. Lane R. D. (2008). Association between trait emotional awareness and dorsal anterior cingulate activity during emotion is arousal-dependent. *NeuroImage* 41 648–655. 10.1016/j.neuroimage.2008.02.030 18406175 PMC2821667

[B48] Menon V. (2015). Salience Network. *Neurosci. Biobehav. Psychol.* 2015 597–611. 10.1016/B978-0-12-397025-1.00052-X

[B49] Meo S. A. Al-Dreess A. M. (2005). Mobile phone related hazards and subjective hearing and vision symptoms in the saudi population. *Int. J. Occupational Med. Environ. Health* 18 45–49. 16052891

[B50] Ming Z. Yang Z. (2007). The relationship between working memory and selective attention. *Adv. Psychol. Sci.* 15 8–15.

[B51] Miri M. Tiyuri A. Bahlgerdi M. Miri M. Miri F. Salehiniya H. (2019). Mobile addiction and its relationship with quality of life in medical students. *Clin. Epidemiol. Global Health* 8 229–232. 10.1016/j.cegh.2019.08.004

[B52] Montag C. Błaszkiewicz K. Sariyska R. Lachmann B. Andone I. Trendafilov B. (2015). Smartphone usage in the 21st century: who is active on WhatsApp? *BMC Res. Notes* 8:331. 10.1186/s13104-015-1280-z 26238512 PMC4522968

[B53] Murphy M. Stickgold R. Parr M. E. Callahan C. Wamsley E. J. (2018). Recurrence of task-related electroencephalographic activity during post-training quiet rest and sleep. *Sci. Rep.* 8:5398. 10.1038/s41598-018-23590-1 PMC5876367 29599462

[B54] Murray M. M. Brunet D. Michel C. M. (2008). Topographic ERP analyses: a step-by-step tutorial review. *Brain Topogr.* 20 249–264. 10.1007/s10548-008-0054-5 18347966

[B55] Naqvi N. H. Rudrauf D. Damasio H. Bechara A. (2007). Damage to the insula disrupts addiction to cigarette smoking. *Science* 315 531–534. 10.1126/science.1135926 17255515 PMC3698854

[B56] Nishida K. Morishima Y. Yoshimura M. Isotani T. Irisawa S. Jann K. (2013). EEG microstates associated with salience and frontoparietal networks in frontotemporal dementia, schizophrenia and Alzheimer’s disease. *Clin. Neurophysiol.* 124 1106–1114. 10.1016/j.clinph.2013.01.005 23403263

[B57] Norbaidurah I. Shazli Ezzat G. Norrafizah J. (2018). Relationship between smartphone addiction with anxiety and depression among undergraduate students in Malaysia. *Int. J. Health Sci. Res.* 8 163–171.

[B58] Pascual-Marqui R. D. Michel C. M. Lehmann D. (1995). Segmentation of brain electrical activity into microstates: model estimation and validation. *IEEE Trans. Biomed. Eng.* 42 658–665. 10.1109/10.391164 7622149

[B59] Perlman S. B. Pelphrey K. A. (2010). Regulatory brain development: balancing emotion and cognition. *Soc. Neurosci.* 5 533–542. 10.1080/17470911003683219 20419567 PMC2950223

[B60] Ruth J. V. H. van den Brink W. Veltman D. J. Goudriaan A. E. (2010). Brain imaging studies in pathological gambling. *Curr. Psychiatry Rep.* 12 418–425. 10.1007/s11920-010-0141-7 20676945 PMC2933850

[B61] Salehan M. Negahban A. (2013). Social networking on smartphones: when mobile phones become addictive. *Comput. Hum. Behav.* 29 2632–2639. 10.1016/j.chb.2013.07.003

[B62] Schlegel F. Lehmann D. Faber P. L. Milz P. Gianotti L. R. R. (2011). EEG microstates during resting represent personality differences. *Brain Topogr.* 25 20–26. 10.1007/s10548-011-0189-7 21644026

[B63] Seeley W. W. Menon V. Schatzberg A. F. Keller J. Glover G. H. Kenna H. (2007). Dissociable intrinsic connectivity networks for salience processing and executive control. *J. Neurosci.* 27 2349–2356. 10.1523/jneurosci.5587-06.2007 17329432 PMC2680293

[B64] Seitzman B. A. Abell M. Bartley S. C. Erickson M. A. Bolbecker A. R. Hetrick W. P. (2017). Cognitive manipulation of brain electric microstates. *NeuroImage* 146 533–543. 10.1016/j.neuroimage.2016.10.002 27742598 PMC5321823

[B65] Soyemi J. Oloruntoba S. A. Okafor B. (2015). Analysis of mobile phone impact on student academic performance in tertiary institution. *Int. J. Emerg. Technol. Adv. Eng.* 5, 361–367.

[B66] Sridharan D. Levitin D. J. Menon V. (2008). A critical role for the right fronto-insular cortex in switching between central-executive and default-mode networks. *Proc. Natl. Acad. Sci. U.S.A.* 105 12569–12574. 10.1073/pnas.0800005105 18723676 PMC2527952

[B67] Takao M. Takahashi S. Kitamura M. (2009). Addictive personality and problematic mobile phone use. *CyberPsychol. Behav.* 12 501–507. 10.1089/cpb.2009.0022 19817562

[B68] Tibshirani R. Walther G. (2005). Cluster validation by prediction strength. *J. Comput. Graph. Stat.* 14 511–528. 10.1198/106186005x59243 12611515

[B69] Tolle T. R. Kaufmann T. Siessmeier T. (1999). Region-specific encoding of sensory and affective components of pain in the human brain: a positron emission tomography correlation analysis. *Ann. Neurol.* 45 40–47. 10.1002/1531-8249(199901)45:1<40::aid-art8>3.0.co;2-l 9894875

[B70] Vinod M. Uddin L. Q. (2010). Saliency, switching, attention and control: a network model of insula function. *Brain Struct. Funct.* 214 655–667. 10.1007/s00429-010-0262-0 20512370 PMC2899886

[B71] Wang Q. Zhang Y. (2015). Relation of mobile phone addiction to perceived social support and subjective well-being in college students. *Chin. Ment. Health J.* 29 868–873.

[B72] Wang Y. Zou Z. Song H. Xu X. Wang H. d’ Oleire Uquillas F. (2016). Altered gray matter volume and white matter integrity in college students with mobile phone dependence. *Front. Psychol.* 7:597. 10.3389/fpsyg.2016.00597 27199831 PMC4855531

[B73] West G. L. Stevens S. A. Pun C. Pratt J. (2008). Visuospatial experience modulates attentional capture: evidence from action video game players. *J. Vision* 8 13–13. 10.1167/8.16.13 19146279

[B74] Wu X. Guo J. Wang Y. Zou F. Guo P. Lv J. (2020). The relationships between trait creativity and resting-state EEG microstates were modulated by self-esteem. *Front. Hum. Neurosci.* 14:576114. 10.3389/fnhum.2020.576114 33262696 PMC7686809

[B75] Xiong J. Zhou Z.-K. Chen W. You Z. Zhai Z.-Y. (2012). Development of the mobile phone addiction tendency scale for college students. *Chin. Ment. Health J.* 26 222–225.

[B76] Zappasodi F. Croce P. Giordani A. Assenza G. Giannantoni N. M. Profice P. (2017). Prognostic value of EEG microstates in acute stroke. *Brain Topogr.* 30 698–710. 10.1007/s10548-017-0572-0 28547185

[B77] Zhang J. Liu Q. (2017). “The influence of smartphone use and addiction on the core components of in College students’ executive function,” *The 20th National Academic Conference on Psychology – Abstracts of Psychology and National Mental Health*, eds Chinese Psychological Association, 498–499.

[B78] Zhang M. (2006). *Get Rid of the Psychological Dependence of Pain–Addiction Psychology.* Beijing: Science Press, 8.

[B79] Zheng X.-Y. Deng L.-Y. (2015). Effects of perceptions of inter-parental conflict and self identity on interent addction in adolescents. *J. Chinese J. Clin. Psychol.* 23 906–910.

[B80] Zhou Z. Zhou H. Zhu H. (2015). Working memory, executive function and impulsivity in Internet-addictive disorders: a comparison with pathological gambling. *Acta Neuropsychiatrica* 28 92–100. 10.1017/neu.2015.54 26400106

